# Characteristics of Interleukin-6 Signaling in Elective Cardiac Surgery—A Prospective Cohort Study

**DOI:** 10.3390/jcm11030590

**Published:** 2022-01-25

**Authors:** Jürgen Puchinger, Sylvia Ryz, Larissa Nixdorf, Maximilian Edlinger-Stanger, Andrea Lassnigg, Dominik Wiedemann, Michael Hiesmayr, Andreas Spittler, Martin H. Bernardi

**Affiliations:** 1 Division of Cardiac Thoracic Vascular Anaesthesia and Intensive Care Medicine, Medical University of Vienna, 1090 Vienna, Austria; juergen.puchinger@gmx.at (J.P.); sylvia.ryz@meduniwien.ac.at (S.R.); maximilian.edlinger-stanger@meduniwien.ac.at (M.E.-S.); andrea.lassnigg@meduniwien.ac.at (A.L.); michael.hiesmayr@meduniwien.ac.at (M.H.); 2 Division for Internal Medicine 3, University Hospital of St. Poelten, Dunant-Platz 1, 3100 Sankt Poelten, Austria; 3 Department of Surgery, Medical University of Vienna, Waehringer Guertel 18-20, 1090 Vienna, Austria; larissa.nixdorf@meduniwien.ac.at (L.N.); andreas.spittler@meduniwien.ac.at (A.S.); 4 Department of Cardiac Surgery, Medical University of Vienna, Waehringer Guertel 18-20, 1090 Vienna, Austria; dominik.wiedemann@meduniwien.ac.at; 5 Core Facilities, Core Facility Flow Cytometry, Medical University of Vienna, 1090 Vienna, Austria

**Keywords:** cardiac surgical procedures, cardiopulmonary bypass, cytokine receptor gp130, inflammation, interleukin-6, receptors, interleukin-6

## Abstract

Interleukin-6 (IL-6) can cause pro- and anti-inflammatory effects via different signaling pathways. This prospective study investigated the perioperative kinetics of IL-6, soluble IL-6 receptor (sIL-6R), and soluble glycoprotein 130 (sgp130) in elective patients undergoing cardiopulmonary bypass (CPB). IL-6, sIL-6R, and sgp130 were measured simultaneously and consecutively at 19 timepoints until the 10th postoperative day (POD). The proportion of pro- and anti-inflammatory pathways were determined by calculating sIL-6R/IL-6 and sIL-6R/sgp130 ratios. We analyzed 93 patients. IL-6 increased during surgery with reaching a plateau two hours after CPB and peaking on POD 1 (188.5 pg mL^−1^ (IQR, 126.6; 309.2)). sIL-6R decreased at the beginning of the surgical procedure, reaching a nadir level on POD 2 (26,311 pg mL^−1^ (IQR, 22,222; 33,606)). sgp130 dropped immediately after CPB initiation (0.13 ng mL^−1^ (IQR, 0.12; 0.15)), followed by a continuous recovery until POD10. The sIL-6R/IL-6 ratio decreased substantially at the beginning of the procedure, reaching a nadir on POD 1 (149.7 (IQR, 82.4; 237.4)), while the sIL-6R/sgp130 ratio increased simultaneously until 6 h post CPB (0.219 (IQR 0.18; 0.27)). In conclusion, IL-6 exhibited high inter-individual variability reflecting an inhomogeneous inflammatory response. Pro-inflammatory effects and overwhelming inflammation were rare and predominantly anti-inflammatory effects were found.

## 1. Introduction

Interleukin- 6 (IL-6) is a widely discussed biomarker in conjunction with extracorporeal circulation and inflammation during cardiac surgical interventions [1,2,3,4,5,6,7].

Beside inflammation, IL-6 is involved in various processes with a complex signaling physiology based on two pathways [8]. In both pathways, a ligand/receptor complex is formed combined with a type I signal transducer protein glycoprotein (gp) gp130 [9].

The classic-signaling process is limited to cells with a membrane-bound IL-6 receptor (IL-6R), which is only found on hepatocytes and several leukocyte subpopulations [10,11]. The IL-6R and its ligand form an IL-6•IL-6R complex, which then associates with membrane-bound gp130. Gp130 is expressed ubiquitously on every cell. The classic-signaling process is associated with regenerative or anti-inflammatory effects [9,10,11,12].

The trans-signaling process is mediated by a soluble form of the IL-6R (sIL-6R) and occurs in all cells expressing gp130. Most of sIL-6R is formed by shedding of membrane-bound IL-6R into the circulation after proteolytic cleavage [13]. Alternatively, some minor fraction of IL-6R is generated by splicing of the IL-6R mRNA [14]. sIL-6R binds free IL-6 and forms IL-6•sIL-6R complexes, which again bind to membrane-bound gp130 and cause IL-6 trans-signaling. Trans-signaling mediates pro-inflammatory effects of IL-6 such as higher endothelial permeability [10,15].

Similar to sIL-6R, a soluble form of gp130 (sgp130) is circulating in plasma [16,17]. Sgp130 is able to bind and neutralize the IL-6•sIL-6R complex and consequently specifically inhibits pro-inflammatory effects [18]. This natural IL-6 buffer is dependent on the sIL-6R levels and is therefore able to antagonize low levels of circulating IL-6 [19]. In physiological conditions, IL-6, sIL-6R, and sgp130 are in equilibrium [9].

This complex IL-6 signaling physiology and balance in pro- and anti-inflammatory effects has been investigated in several studies concerning cardiac patients. Imbalances were found in patients with severe chronic heart failure [20], in the local and circadian inflammatory reaction in the coronary blood stream of the ischemic heart [21,22]. Moreover, a small study in 31 patients investigated the effects of cardiopulmonary bypass (CPB) on IL-6, sIL-6R, and sgp130 in patients undergoing coronary artery bypass graft [7].

The aim of this study was to investigate the perioperative kinetics of IL-6, sIL-6R, and sgp130 in order to characterize pro- and anti-inflammatory IL-6 effects and the natural buffer system in a close-meshed perioperative timeframe in patients undergoing elective cardiac surgery with cardiopulmonary bypass (CPB). Additionally, we investigated the association between the changes in the balance between pathways/mediators and perioperative fluid balance and cell membrane integrity.

## 2. Materials and Methods

In this prospective, observational, single-centre study, we included 100 elective cardiac surgical patients with planned CPB. The study was performed at the Division of Cardiac Thoracic Vascular Anaesthesia and Intensive Care Medicine at Medical University of Vienna and conducted between 31 October 2016, and 26 January 2018.

We excluded patients who were less than 18 years of age, patients who were pregnant, patients with a body mass index <18, C-reactive protein >20 mg L^−1^, and patients with pre-operative chronic renal failure on renal replacement therapy. Moreover, we excluded patients who were receiving chemotherapy or diagnosed with any disease state associated with leukopenia, receiving anti-leucocyte drugs, immunosuppression, or TNF- α blockers. Patients undergoing emergency procedures, transplantation surgery, pulmonary thromboendarterectomy, and elective cardiac assist device implantation or patients receiving postoperative extracorporeal membrane oxygenation were also excluded. Finally, patients who did not provide their written informed consent to participate were excluded.

This study was approved by the Ethics committee (Ref: 1518/2016) of the Medical University of Vienna, Vienna, Austria (chairperson: Dr. Jürgen Zezula) on 7 July 2016. Written informed consent was obtained from all the subjects participating in the trial.

### 2.1. Outcome Variables

The primary outcome was the change in IL-6, sIL-6R, and sgp130 levels in the perioperative course. As secondary outcomes, we investigated the change in sIL-6R/IL6 and sIL-6R/sgp130 ratio levels in the perioperative course, changes in intra- and postoperative fluid balances, and differences in postoperative phase angle, a marker of cell membrane integrity measured by bioelectrical impedance analysis (BIA).

### 2.2. Procedure, Data and Sample Collection

We collected pre-operative patient data, comorbidities, surgery- and procedure-related factors, and post-operative data (Table 1). Data collection was performed with the help of a case report form.

Patients were enrolled the day before surgery after giving informed consent. Patient data were collected prospectively at the time of enrolment and followed until their hospital discharge or for a maximum of 10 days.

Blood sample time points:

1. After induction of anaesthesia-before skin incision
2. Before start of CPB-after sternotomy/thoracotomy
3. 30 min after start of CPB
4. 120 min after start of CPB
5. After end of CPB-before protamine administration
6. 60 min after end of CPB
7. 120 min after end of CPB
8. 240 min after end of CPB
9. 360 min after end of CPB
–19. Postoperative Day (POD) 1–10

Blood samples were taken from central venous lines in all patients until the line was removed or the patients were discharged from hospital. Blood samples were drawn in pyrogen-free vials, centrifuged at 3000 rpm and the resultant supernatant was immediately stored frozen at −80 °C for further analysis in several aliquots. Quantitative measurements of IL-6, sIL-6R, and sgp130 concentrations were conducted with 96-well plate ELISAs (Human IL-6 Quantikine ELISA Kit, Human IL-6R alpha Quantikine ELISA Kit and Human soluble gp130 Quantikine ELISA Kit, R&D Systems^®^ Inc., Minneapolis, MN, USA), according to the manufacturer’s protocol. The reported coefficients of variability in the used ELISA Kits are 4.2%, 8.6%, and 5.5% for intra-assay precision and 6.4%, 6.4%, and 5.2% for inter-assay precision, respectively.

### 2.3. Bioelectrical Impedance Analysis

We performed BIA by using 800 μA at 50 kHz with a single-frequency bioimpedance analyzer (Model BIA 101; Akern-RJL, Pontassieve, Italy). The skin was cleaned, and adhesive pre-gelled electrodes (Bianostic AT; Data Input GmbH, Wedemark, Germany) were placed on the hand and the foot of the right side while patients where in a recumbent position with the limbs abducted from the body. Measurements were performed preoperatively before induction of anaesthesia and once daily postoperatively until POD 10 or hospital discharge. The measured BIA variables were resistance (R), reactance (Xc) and the phase angle (arctanXc/R).

### 2.4. Statistics

Demographic and clinical baseline data are presented as mean and standard deviation (SD) or median with inter-quartile range (IQR) for metric variables and absolute frequencies for categorical variables. Differences between groups were analysed using the Student’s *t*-test for normally distributed variables and the Mann–Witney U-test for non-normally distributed continuous variables. The Shapiro–Wilk test was used to test normality. The χ^2^ test was used to test categorical variables. A paired *t*-test was used to compare dependent samples, autocorrelation was used to test the time dependency and correlation of serial measurements.

To analyse the difference in clinical effects (perioperative fluid balance and postoperative BIA) of pro-inflammatory IL-6, we selected the period of increasing inflammatory reaction beginning with the start of CPB up to 6 h after CPB (timepoints 3–9). Within this period, we divided all patients to deciles and formed 3 different groups: patients with (a) continuously highest decile; (b) continuously lowest decile; (c) and continuously between lowest and highest (intermediate) perioperative sIL6-R/IL-6 ratio. Since sIL6-R/IL-6 ratios were changing dynamically within the observed time period, we determined that patients had to be at least 50% or more of timepoints within the highest or lowest sIL6-R/IL-6 ratios to be selected to the respective group.

The consecutive emerging clinical effects of inflammation were analysed by differences between these groups in the postoperative course. For the non-parametric longitudinal testing of differences within the respective groups, we used the Friedman test.

A *p*-value of <0.05 was considered statistically significant. No adjustment for multiple testing was performed. Statistical analysis was performed, and plots were drawn using the statistical environment R 3.4.3 (http://www.R-project.org/).

## 3. Results

### 3.1. Patient Characteristics

**Figure 1:**
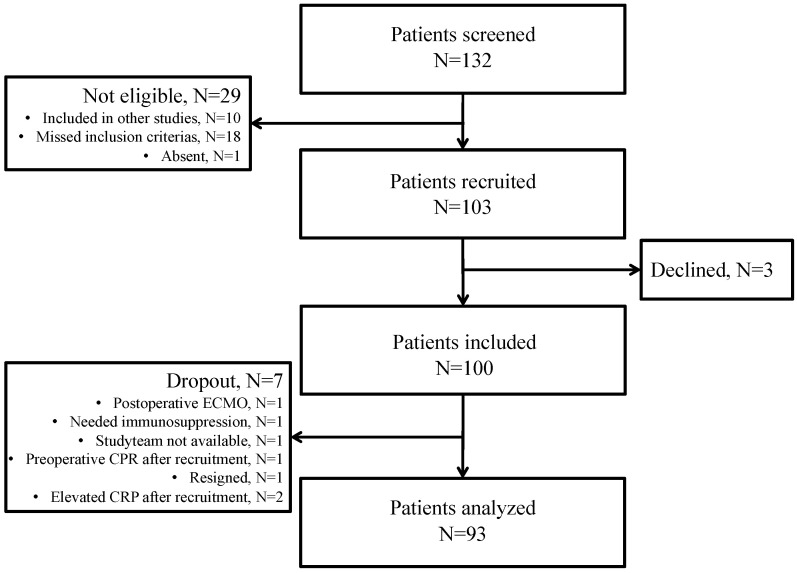
Selection and exclusion criteria for patients enrolled to the study.

A total of 132 patients were screened for eligibility. Of these, 29 patients were excluded (Figure 1). We approached 103 patients, and three of those declined informed consent. After including 100 patients, seven dropped out. Finally, 93 patients were included in the analysis.

The median age of our patients was 69.0 years (IQR 60.0 to 76.0 years), and 37% (*n* = 34) were females (Table 1). CABG, valve, and combined procedures were performed in 15% (*n* = 14), 57% (*n* = 53), and 28% (*n* = 26) of patients, respectively. The median procedure time was 307 min (IQR 255–542 min). A simplified acute physiology score (SAPS) 3 of 41.5 (IQR 34.8–49.0) was found after admission to intensive care unit (ICU) and the median length of ICU stay was two days (IQR 1–4 days). Follow-up of blood samples was decreased on postoperative day (POD) 4. Either patients’ central venous lines were removed, or patients were consecutively discharged from hospital.

### 3.2. Laboratory Measurements

**Figure 2:**
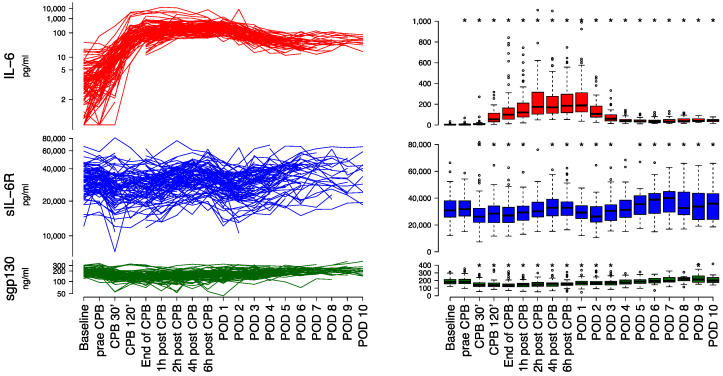
Exaggeration of IL-6, sIL-6R and sgp130. The figure displays individual time course (**left**) and corresponding boxplots (**right**) of perioperative levels of IL-6, sIL-6R and sgp130 levels. The lines on the left are displayed on a a logarithmic scale. Asterisks (*) mark significant differences compared to baseline at *p* less than 0.05. In the boxplots, the lower boundary of the box indicates the 25th percentile, a black line within the box marks the median, and the upper boundary of the box indicates the 75th percentile. Whiskers above and below the box indicate the 10th and 90th percentiles. Points (°) above and below the whiskers indicate outliers outside the 10th and 90th percentiles (IL-6 outliers above 1200 pg/mL are not shown). Red, blue and green lines indicate IL-6, sIL-6R and sgp130 levels, respectively. Abbreviations: CPB, cardiopulmonary bypass; POD, postoperative day; sgp130, soluble glycoprotein 130; sIL-6R, soluble interleukin-6 receptor.

IL-6: Median baseline IL-6 was 3.0 pg mL^−1^ (IQR 2.0–4.3 pg mL^−1^). IL-6 increased continuously during the procedure with reaching a plateau two to six hours after CPB with highest median IL-6 concentration on POD 1 (188.5 pg mL^−1^ (IQR 126.6–309.2 pg mL^−1^)). The mean difference between POD 1 and baseline was 256.5 pg mL^−1^ (95% CI, 209.8–303.1 pg mL^−1^; *p* < 0.0001). The decline of IL-6 started on POD 2 and was maintained until the end of observation period, not returning to baseline values (Figure 2 and Supplementary Figure S1). The sequentially measured IL-6 values had a positive autocorrelation of 0.76 and 0.51 one timepoint and two timepoints prior, respectively.

sIL-6R: Median baseline sIL-6R was 30,859 pg mL^−1^ (IQR 25,874–37,980 pg mL^−1^). sIL-6R significantly decreased from baseline to a nadir 30 min after CPB start of 26,161 pg mL^−1^ (IQR 22,151 to 32,408 pg mL^−1^) with a mean difference of −4083.7 pg mL^−1^ (95% CI, −5674.0–−2493.4 pg mL^−1^; *p* = 0.0019) compared to baseline. This was followed by an increase until 6 h after CPB, with a mean difference of 5794.2 pg mL^−1^ (95% CI, 3756.3–7832.1pg mL^−1^; *p* = 0.0002) compared to 30 min after CPB start. This increase was followed by a continuous decrease on POD 1 and POD 2, with a mean difference of −4205.4 pg mL^−1^ (95% CI, −5588.2–−2822.6 pg mL^−1^; *p* < 0.0001) and −5930.6 pg mL^−1^ (95% CI, −7801.1–−4060.1 pg mL^−1^; *p* < 0.0001) compared to 6 h after CPB, respectively. This was followed with a consecutive recovery until POD 4 (Figure 2). The sequentially measured sIL-6R values had a positive autocorrelation of 0.75 and 0.67 one timepoint and two timepoints prior, respectively.

sgp130: Median baseline sgp130 was 179.6 ng mL^−1^ (IQR 157.9–213.1 ng mL^−1^). sgp130 significantly decreased from baseline to a nadir at the end of CPB of 133.7 ng mL^−1^ (IQR 117.7 to 153.7 ng mL^−1^) with a mean difference of −42.8 ng mL^−1^ (95% CI, −50.0–−35.6 ng mL^−1^; *p* < 0.0001) compared to baseline. This decrease was followed by a continuous recovery until the end of the observational period (Figure 2). The sequentially measured sgp130 values had a positive autocorrelation of 0.72 and 0.63 one timepoint and two timepoints prior, respectively.

**Figure 3:**
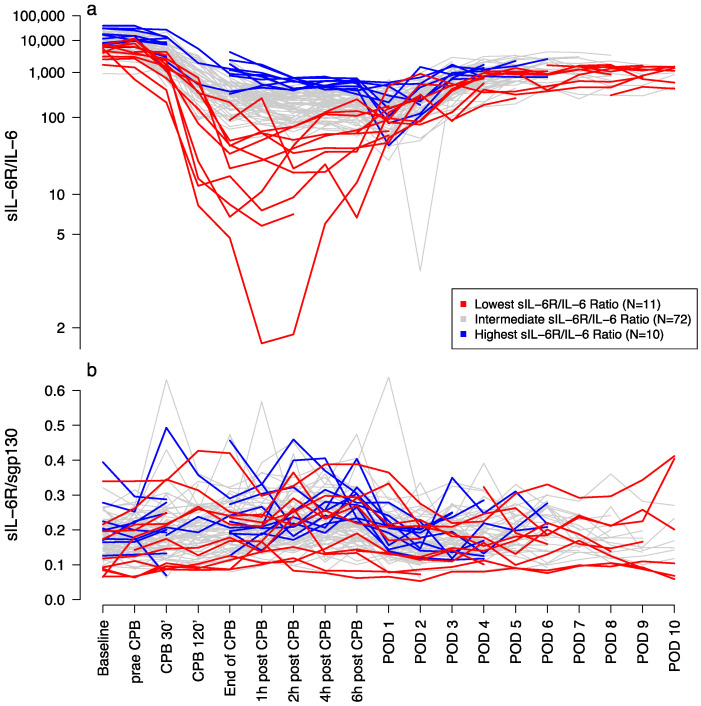
sIL-6/IL-6 and sIL-6R/sgp130 ratio. The lines show the perioperative levels of (**a**) sIL-6/IL-6 and (**b**) sIL-6R/sgp130 ratio at different time points. Red lines indicate patients with continuously lowest levels, blue lines indicate patients with continuously highest levels and grey lines indicate patients with intermediate levels of perioperative sIL6-R/IL-6 ratio measured between timepoint 3 and 9. Abbreviations: CPB, cardiopulmonary bypass; POD, postoperative day; sgp130, soluble glycoprotein 130; sIL-6R, soluble interleukin-6 receptor.

sIL-6R/IL-6 ratio: Median baseline sIL-6R/IL-6 ratio was 9870.0 (6340.0–16,323.7). The ratio decreased substantially from baseline to 120 min after start of CPB with a mean difference of −10,831.38 (95% CI, −12,660.1–−9002.7; *p* < 0.0001). The nadir of 149.7 (IQR 82.4–237.4) was found on POD 1, followed by a consecutive increase until the end of the observational period, not reaching the baseline ratio (Figure 3a).

sIL-6R/sgp130 ratio: Median baseline sIL-6R/sgp130 ratio was 0.17 (IQR 0.14–0.21). The ratio increased from baseline to a peak of 0.22 (IQR 0.18–0.17) six hours after the end of CPB with a mean difference of 0.05 (95% CI, 0.04–0.06; *p* < 0.0001). On POD 1, the sIL-6R/sgp130 ratio decreased again to 0.18 (IQR 0.14–0.21), with a mean difference of −0.04 (95% CI, −0.05–−0.03; *p* < 0.0001) followed by an increase until the end of the observational period (Figure 3b)

### 3.3. Pro-Inflammtory Effects on Fluid Balance and BIA

We found a more pronounced decrease of sIL-6R/IL-6 ratio in patients with high IL-6 levels during this period (Figure 3a). Those patients with the continuously lowest sIL-6R/IL-6 ratios were also found with a significant steady increase of sIL-6R/sgp130 ratios (mean difference, 0.03, 95% CI 0.003–0.066; *p* = 0.0352) until POD 1 (Figure 3b), whereas those patients with the continuously highest sIL-6R/IL-6 ratios had no change in their sIL-6R/sgp130 ratios (mean difference −0.01, 95% CI −0.07–0.05; *p* = 0.6998) until POD 1.

**Figure 4:**
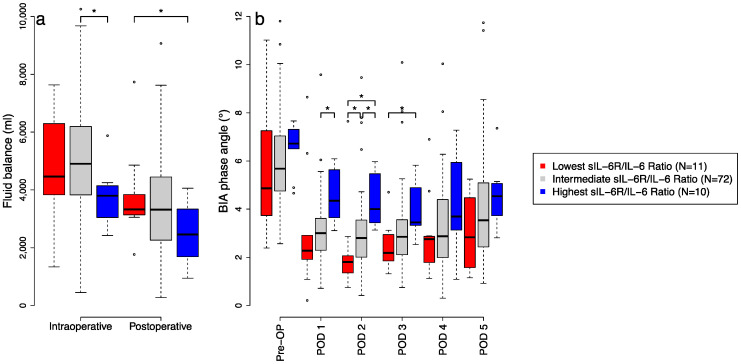
Effects of inflammation on fluid balance and BIA. (**a**) The boxplots show differences in intra- and postoperative fluid balance between patients with continuously highest levels (blue), intermediate levels (grey) and lowest levels (red) of perioperative sIL6-R/IL-6 ratio measured between timepoint 3 and 9. (**b**) The boxplots show differences in phase angle between patients with continuously highest levels (blue), intermediate levels (grey) and lowest levels (red) of perioperative sIL6-R/IL-6 ratio measured between timepoint 3 and 9. Asterisks (*) mark significant differences between the two groups at *p* less than 0.05. In the boxplots, the lower boundary of the box indicates the 25th percentile, a black line within the box marks the median, and the upper boundary of the box indicates the 75th percentile. Whiskers above and below the box indicate the 10th and 90th percentiles. Points (°) above and below the whiskers indicate outliers outside the 10th and 90th percentiles. Abbreviations: BIA, bioelectrical impedance analysis; CPB, cardiopulmonary bypass; POD, postoperative day; sIL-6R, soluble interleukin-6 receptor.

We found a significantly lower cumulative fluid balance (i.e., induction of anaesthesia until first postoperative morning) on the day of surgery between the high and low sIL-6R/IL-6 ratio group, namely 6166 ± 1161 mL vs. 8597 ± 2507 mL, *p* = 0.0159, respectively, as well as in patients in the high sIL-6R/IL-6 group compared to the intermediate group, 6166 ± 1161 mL vs. 8472 ± 3152 mL, *p* = 0.0082, respectively (Figure 4a). The difference in fluid balance was mainly caused by a reduced fluid resuscitation in the first postoperative hours in ICU after end of surgery until POD 1.

The variability of median phase angle levels within the group with lower and higher sIL-6R/IL-6 ratios was not significantly different over the postoperative period (*p* = 0.9869 and *p* = 0.8088, respectively).

In the group with lower sIL-6R/IL-6 ratios, postoperative phase angle was significantly lower on POD 2 (2.3 ± 2.0 vs. 5.4 ± 3.7; *p* = 0.0015) and POD 3 (2.5 ± 1.0 vs. 4.0 ± 1.2; *p* = 0.0046) compared to the high sIL-6R/IL-6 ratio group (Figure 4b). Because of the frequent discharge of patients after POD 5, BIA measurements after POD 5 were not taken into account.

We found a significantly higher frailty scale (3.0 (2.0; 5.0) vs. 1.0 (1.0; 2.0); *p* = 0.0048), procedure time (397 min (3461; 498) vs. 240 min (202; 298); *p* = 0.0003), higher need for transfusions (2 units (1; 3) vs. no transfusions; *p* = 0.0005), a higher SOFA on ICU admission (9.0 (8.5; 9.0) vs. 6.0 (5.0; 7.0); *p* = 0.0004), and a significantly longer ICU-length of stay (5.0 days (2.5; 9.0) vs. 1 day (1.0; 1.0); *p* = 0.0002) in patients within the lower sIL-6R/IL-6 ratio group compared to patients within the high sIL-6R/IL-6 ratio group, respectively (Supplementary Table S1).

## 4. Discussion

Cardiac surgery is associated with an unpredictable activation of the immune system, which is mainly caused by blood contact with artificial surfaces, shear forces from roller pumps, and surgical trauma [6,23]. In this clinical analysis, we describe the perioperative kinetics of IL-6, sIL-6R, and sgp130 in a close meshed real-life observation of a large cohort of elective cardiac surgical patients.

We observed an elevation of IL-6 after the initiation of CPB reaching a plateau at the end of surgery until the first postoperative day. Moreover, we observed an inhomogeneous inflammatory response and strong inter-individual differences in the amounts of IL-6 between our patients, similar to previous findings [6,7]. Interestingly, Corbi et al. [7] found in a pilot study a higher inflammatory reaction in off-pump patients than in on-pump CABG patients, meaning the inflammatory activation is mainly caused by surgical trauma, not by CPB, a fact we cannot confirm due to no off-pump patients in our patient cohort. In addition, we can show a more accurate, close-meshed time frame in a higher number of patients.

Nevertheless, the overall activation of IL-6 was below a hyperinflammatory or septic response, where persistently elevated levels of IL-6 greater than 500 pg mL^−1^ can be found [24,25].

We found a high quantity of sIL-6R preoperatively, which is in line with reported physiologic quantities of sIL-6R being 10,000 times higher than IL-6 [11,26]. Therefore, the perioperative sIL-6R concentrations are relatively stable throughout the perioperative course in cardiac surgical patients and an inverse course to IL-6 changes can be observed, especially in the beginning of the inflammatory response.

Consistent with current literature, we found more than five times higher levels of sgp130 than sIL-6R [19,27]. Such high concentrations of sgp130 are necessary since the natural inhibition of the IL-6•sIL-6R complex only works if molar levels of sgp130 are above molar levels of sIL-6R [15,18].

Appropriately, we observed an immediate decrease of sgp130 when IL-6 starts to increase. This finding supports the currently discussed hypothesis [7,19] of sgp130 as instant neutralizer of low levels of circulating IL-6. However, hemodilution due to CPB-priming has to be considered as factor involved lowering sgp130 levels.

For a better understanding of the proportion of pro- and anti-inflammatory effects, we estimated sIL-6R/IL6 and sIL-6R/sgp130 ratios [15]. Pro-inflammatory effects result in a decrease of the sIL-6R/IL-6 ratio, and subsequently, the sgp130-buffering results in an increase of the sIL-6R/sgp130 ratio. If pro- and anti-inflammation are in balance, it can be assumed that these ratios remain unchanged.

We showed a substantial decrease of sIL-6R/IL-6 ratio as marker for increasingly formed IL-6•sIL-6R complexes suggesting an increase in pro-inflammatory effects. From in vitro and animal studies it is known that pro-inflammatory properties of IL-6 are mediated by the IL-6•sIL-6R complex [9,15]. In our study, this substantial decrease of the sIL-6R/IL-6 ratio is caused by two factors. First, at the beginning of the surgical procedure the concentration of free IL-6 increased and thereby caused a decrease of the ratio. Secondly, due to the increase of free IL-6, the concentration of free sIL-6R decreased as it naturally forms complexes with free IL-6. Simultaneously, the sIL-6R/sgp130 ratio increased, which can be seen as marker for the buffering of IL-6•sIL-6R complexes and therefore neutralizing and blocking of the pro-inflammatory pathway. Further, we found a drop in sIL-6R on POD 1 and POD 2, which can be a result of forming IL-6•sIL-6R complexes with increasing sIL-6R/sgp130 ratio.

It might be argued that these increasingly formed IL-6•sIL-6R complexes and increasing sIL-6R/sgp130 ratio caused IL-6 trans-signaling and consequently pro-inflammatory symptoms in our patients. Our patients with suspected high pro-inflammatory activity (high trans-signaling activity, low sIL-6R/IL-6 ratio) were older, frailer, and had a more complicated intraoperative course.

Furthermore, we observed with a suspected high pro-inflammatory activity a significantly higher postoperative fluid balance. The activation of the IL-6 pro-inflammatory pathway leads to higher endothelial permeability [12,28]. Interestingly, in those patients with suspected high pro-inflammatory activity, we found also consistently lower phase angles in the first postoperative days, which may be a consequence of decreased cell membrane integrity [29]. Since extra- and intracellular fluid shifts are also reflected in phase angle changes [30], these lower phase angles also explain the observed pro-inflammatory fluid redistribution. Nevertheless, lower phase angles are associated with mortality in critically ill patients [31]. However, the number of patients was too low to confirm this hypothesis.

A low sIL-6R/IL-6 ratio can be achieved by either low amounts of sIL-6R or high amounts of IL-6. The levels of sIL-6R are influenced by a single-nucleotide polymorphism (SNP, rs2221845), which causes a two-times upregulation of sIL-6R [32,33,34]. It has been shown by several authors that this SNP has serious consequences regarding susceptibility to inflammatory diseases, such as coronary heart disease or diabetes [19,34,35]. Nevertheless, we did not find differences in IL-6 or IL-6R baseline levels in patients with coronary heart disease compared to patients with structural heart disease, nor in patients with diabetes.

A recent study by Scheller and colleagues [12] experimentally described the balance of IL-6, sIL-6R, and IL-6•sIL-6R•sgp130 complexes in an in vitro model. The authors concluded that, systemic levels of sgp130 were not sufficient to inhibit pro-inflammatory effects. However, they also stated that IL-6•sIL-6R and IL-6•sIL-6R•sgp130 complexes were formed with much lower frequency than previously thought, which to their argumentation leaves more space for classic-signaling than for trans-signaling (i.e., anti-inflammatory effects predominate pro-inflammatory effects). Nevertheless, it is still unclear, which of the IL-6 signaling pathways result in real functional consequences in vivo [11].

We similarly showed that although systemic IL-6 is substantially increasing at the beginning of surgery, the sIL-6R/sgp130 ratio is only increasing slowly, which supports the argumentation that there is a higher activation of anti-inflammation than of pro-inflammation. Consecutively, we showed that hyperinflammatory states following CPB are rare.

### Limitations

First, we did not measure IL-6•sIL-6R complexes. Only ratios were calculated. This limits our findings to hypothetical interpretations.

Second, our patients underwent elective surgery. Consequently, overwhelming inflammation and complications were rare. It is reported that IL-6 plasma levels can be elevated to the 100–1000 ng mL^−1^ range [13]. By contrast, we found the highest concentrations of 13,000 pg mL^−1^. As a result, we could not make any statements concerning sgp130, sIL-6R, and IL-6 serum concentrations in patients with postoperative complications, such as sepsis or serious infections.

Third, effect modifications owing to omitted or unobserved confounding risk indicators cannot be excluded, although we included the most relevant risk indicators to rule out any systematic effect. However, we did not monitor the use of non-steroidal anti-inflammatory drugs preoperatively (e.g., aspirin), statins, or metformin, which may have some anti-inflammatory effects [36,37].

## 5. Conclusions

We observed typical perioperative IL-6 kinetics in patients undergoing cardiac surgery with CPB. Moreover, we showed the mechanisms of pro- and anti-inflammatory pathways, the perfectly working natural buffer in a real-life environment, and that overwhelming inflammation is rare. The paradigm of a naturally formed buffer for IL-6 by sIL-6R and sgp130 is strongly supported by our findings. Therefore, our data suggest that systemic free IL-6 causes anti-inflammatory effects via membrane-bound IL-6R rather than pro-inflammatory effects via sIL-6R in elective cardiac surgical patients undergoing CPB. Therefore, measures to reduce anti-inflammatory reactions are not needed.

## Figures and Tables

**Table 1:** Demographic and surgical characteristics.

Preoperative Risk Indicators	
Male	59 (63.4)
Female	34 (36.6)
Age (y)	69.0 [60 to 76]
Height (cm)	172 [165 to 178]
Weight (kg)	81.0 [69 to 88]
Resistance	386.5 [345 to 463]
Reactance	43.0 [34.0 to 50.8]
Phase angle	5.7 [4.7 to 7.2]
Frailty scale	2.0 [2 to 3]
Comorbidities	
Asthma	4 (4.3)
COPD	15 (16.1)
NIDDM	14 (15.1)
IDDM	4 (4.3)
Chronic kidney disease	6 (6.5)
Cardiac decompensation	1 (1.1)
PAOD	6 (6.5)
Atrial fibrillation	26 (28)
Angina pectoris	
Absent	68 (73.1)
Stable	23 (24.7)
Unstable	2 (2.2)
LVEF	
>50%	65 (69.9)
30–50%	23 (24.7)
<30%	5 (5.4)
Surgical characteristics	
Procedure	
CABG	14 (15.1)
Combined	26 (28)
Valve	53 (57)
Reoperation	16 (17.2)
Anaesthesia duration (min)	395 [339 to 457]
Surgery (min)	307 [255 to 542]
CPB (min)	148 [111 to 192]
AoCC (min)	98 ± 44
Balance_intraoperative_ (mL)	4460 [3767 to 6096]
PRBC (units)	0 [0 to 1]
Platelets (units)	0 [0 to 0]
Fresh frozen plasma (units)	0 [0 to 0]
Fibrinogen (g)	0 [0 to 2]
Coagulation factors (I.U.)	0 [0 to 0]
Postoperative risk indicators	
SAPS 3	41.1 ± 11.6
SOFA on ICU admission	7 [6 to 9]
Length of ICU stay (d)	2 [1 to 4]

Values are presented as number (*n*) and percentage (%), mean ± standard deviation for normally distributed or median [interquartile range] for non-normally distributed. Abbreviations: AoCC, aortic cross-clamp; CABG, coronary artery bypass graft; COPD, chronic obstructive pulmonary disease; CPB, cardiopulmonary bypass; ICU, intensive care unit; IDDM, insulin-dependent diabetes mellitus; LVEF, left ventricular ejection fraction; NIDDM, non-insulin-dependent diabetes mellitus; PAOD, peripheral artery occlusive disease; PRBC, packed red blood cells; SAPS, simplified acute physiology score; SOFA, sepsis-related organ failure assessment score.

## Data Availability

The data that support the findings of this study are available in anonymized form from the corresponding author on reasonable request and after agreement with the local ethics committee.

## Supplementary Materials

The following supporting information can be downloaded at: https://www.mdpi.com/article/10.3390/jcm11030590/s1, Figure S1: Exaggeration of IL-6. The box plots show the perioperative levels of IL-6 at different time points. In the box plots, the lower boundary of the box indicates the 25th percentile, a black line within the box marks the median, and the upper boundary of the box indicates the 75th percentile. Whiskers above and below the box indicate the 10th and 90th percentiles. Points above and below the whiskers indicate outliers outside the 10th and 90th percentiles. Abbreviations: CPB, cardiopulmonary bypass; POD, postoperative day; sgp130, soluble glycoprotein 130; sIL-6R, soluble interleukin-6 receptor, Table S1: Characteristics in patients with high vs. low sIL-6R/IL-6 ratio.
