# Supplementary material for: Characteristics of Interleukin-6 Signaling in Elective Cardiac Surgery—A Prospective Cohort Study
Source: J Clin Med. 2022 Jan 25;11(3):590. doi: 10.3390/jcm11030590 (PMC8836792; doi:10.3390/jcm11030590)
Supplement: Supplementary file 1 [file jcm-11-00590-s001.zip › Table S1.pdf]

Supplemental Table S1. Demographic characteristics in patients with high vs. low sIL−6R/IL−6 ratio

	Low sIL−6R/IL−6 Ratio	High sIL−6R/IL−6 Ratio	p-value
**Preoperative risk indicators**			
Male	3	7	0.1284
Female	8	3	
Age (y)	72 [68; 77]	56 [51; 61]	0.0005
Height (cm)	164 [156; 175]	175 [169; 178]	0.0615
Weight (kg)	72.0 [63.0; 83.0]	92.0 [83.9; 105.8]	0.0527
Resistance	380 [372; 463]	369 [331; 399]	0.5730
Reactance	35 [26; 51]	44 [36; 51]	0.4806
Phase angle	4.9 [3.7; 7.3]	6.7 [6.5; 7.3]	0.2816
Frailty scale	3.0 [2.0; 5.0]	1.0 [1.0; 2.0]	0.0048
**Comorbidities**			
Asthma	1 (9)	0 (0)	1.0
COPD	3 (27)	3 (30)	1.0
NIDDM	2 (18)	1 (10)	1.0
IDDM	0 (0)	0 (0)	1.0
Chronic kidney disease	0 (0)	1 (10)	0.961
Cardiac decompensation	0 (0)	0 (0)	1.0
PAOD	2 (18)	1 (10)	1.0
Atrial fibrillation	4 (36)	0 (0)	0.118
Angina pectoris			
Absent	10 (91)	7 (70)	
Stable	0 (0)	3 (30)	0.1058
Unstable	1 (9)	0 (0)	
LVEF			
>50%	5 (46)	9 (90)	
30-50%	5 (46)	1 (10)	0.09196
<30%	1 (9)	0 (0)	
**Surgical characteristics**			
Procedure			
CABG	0 (0)	3 (30)	
Combined	5 (46)	2 (20)	0.1142
Valve	6 (55)	5 (50)	
Reoperation	4 (36)	2 (20)	0.7298
Anaesthesia duration (min)	486 [431; 592]	322 [312; 390]	0.0003
Surgery (min)	397 [3461; 498]	240 [202; 298]	0.0003
CPB (min)	176 [144; 225]	107 [93; 126]	0.0025
AoCC (min)	89 [68; 85]	76 [62; 85]	0.3443
$Balance_{intraoperative}$ (ml)	4460 [3832; 6294]	3794 [3109; 4091]	0.1321
PRBC (units)	2 [1; 3]	0 [0; 0]	0.0005
Platelets (units)	0 [0; 1]	0 [0; 0]	0.3279
Fresh frozen plasma (units)	0 [0; 0]	0 [0; 0]	0.3908
Fibrinogen (g)	2 [0; 2]	0 [0; 0]	0.0036
Coagulation factors (I.U.)	0 [0; 1250]	0 [0; 0]	0.0575
**Postoperative risk indicators**			
SAPS 3	52 [41; 53]	36 [34; 39]	0.0835
SOFA on ICU admission	9.0 [8.5; 9.0]	6.0 [5.0; 7.0]	0.0004
Length of ICU stay (d)	5.0 [2.5; 9.0]	1 [1.0; 1.0]	0.0002

Values are presented as number (n) and percentage (%), or median (interquartile range). Abbreviations: AoCC, aortic cross-clamp; CABG, coronary artery bypass graft; COPD, chronic obstructive pulmonary disease; CPB, cardiopulmonary bypass; ICU, intensive care unit; IDDM, insulin-dependent diabetes mellitus; LVEF, left ventricular ejection fraction; NIDDM, non-insulin-dependent diabetes mellitus; PAOD, peripheral artery occlusive disease; PRBC, packed red blood cells; SAPS, simplified acute physiology score; SOFA, sepsis-related organ failure assessment score.
