# Supplementary figures and images for: Characteristics of Interleukin-6 Signaling in Elective Cardiac Surgery—A Prospective Cohort Study
Source: J Clin Med. 2022 Jan 25;11(3):590. doi: 10.3390/jcm11030590 (PMC8836792; doi:10.3390/jcm11030590)

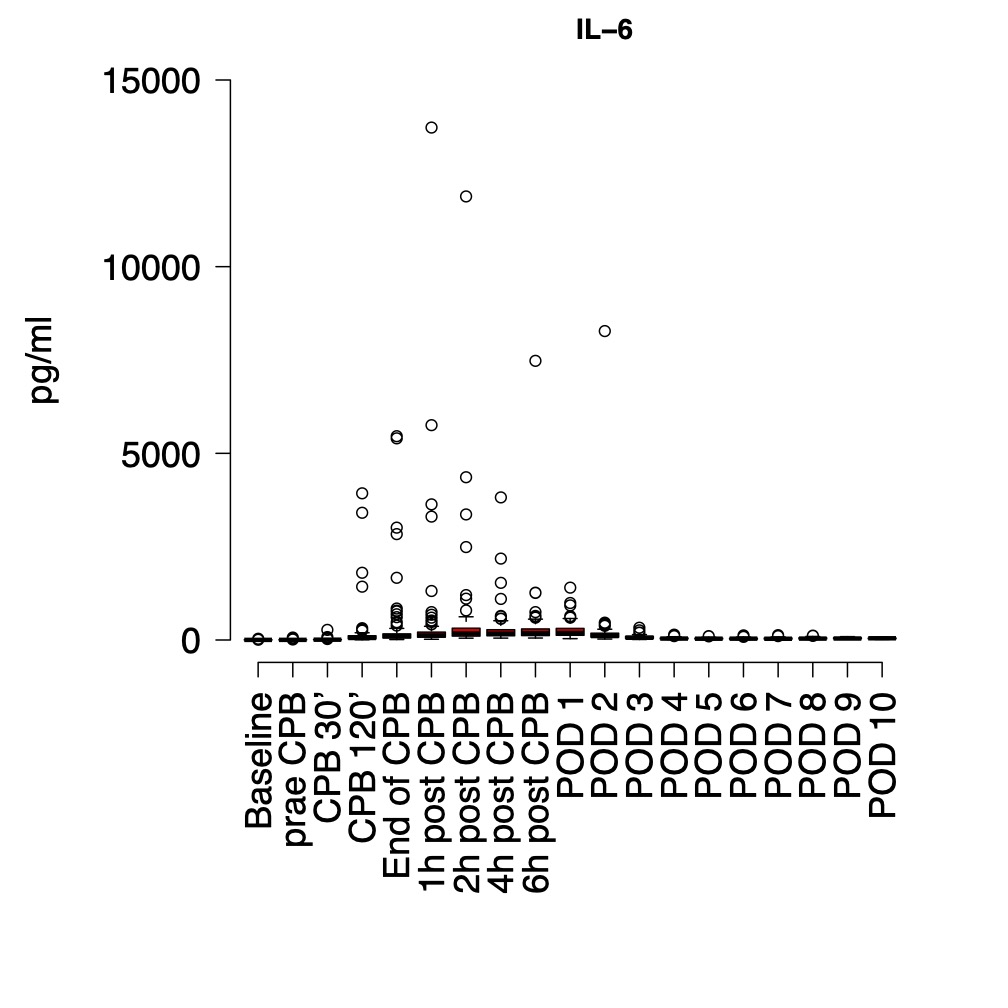

Supplement: Supplementary file 1 [file jcm-11-00590-s001.zip › Figure S1.jpg]
